# Smartphone-Based Contingency Management Intervention to Improve Pre-Exposure Prophylaxis Adherence: Pilot Trial

**DOI:** 10.2196/10456

**Published:** 2018-09-10

**Authors:** John T Mitchell, Sara LeGrand, Lisa B Hightow-Weidman, Mehri S McKellar, Angela DM Kashuba, Mackenzie Cottrell, Tony McLaurin, Goutam Satapathy, F Joseph McClernon

**Affiliations:** 1 Department of Psychiatry and Behavioral Sciences Duke University Medical Center Durham, NC United States; 2 Duke Center for Addiction Science and Technology Durham, NC United States; 3 Center for Health Policy and Inequalities Research at Duke University Duke Global Health Institute Durham, NC United States; 4 Institute of Global Health and Infectious Diseases University of North Carolina at Chapel Hill Chapel Hill, NC United States; 5 Division of Infectious Diseases Duke University Medical Center Durham, NC United States; 6 Division of Pharmacotherapy and Experimental Therapeutics UNC Eshelman School of Pharmacy University of North Carolina at Chapel Hill Chapel Hill, NC United States; 7 Intelligent Automation Incorporated Rockville, NC United States

**Keywords:** HIV, preexposure prophylaxis, mobile health

## Abstract

**Background:**

Pre-exposure prophylaxis (PrEP) provides a strong preventative benefit to individuals at risk for HIV. While PrEP adherence is highly correlated with its efficacy, adherence rates are variable both across and within persons.

**Objective:**

The objective of this study was to develop and pilot-test a smartphone-based intervention, known as mSMART, that targets PrEP adherence. mSMART provides contingency management in the form of monetary incentives for daily PrEP adherence based on a real-time adherence assessment using a camera-based medication event-monitoring tool as well as medication reminders, PrEP education, individualized behavioral strategies to address PrEP adherence barriers, and medication adherence feedback.

**Methods:**

This was a 4-week open-label, phase I trial in a community sample of young men who have sex with men already on PrEP (N=10).

**Results:**

Although adherence composite scores corresponding to PrEP biomarkers indicated that 90% (9/10) of the sample already had an acceptable baseline adherence in the protective range, by the end of the 4-week period, the scores improved for 30% (3/10) of the sample—adherence did not worsen for any participants. Participants reported mean PrEP adherence rates of 91% via daily entries in mSMART. At the end of the 4-week period, participants indicated acceptable ratings of satisfaction, usability, and willingness to recommend mSMART to others. There were no technical difficulties associated with smartphone compatibility, user misunderstandings about mSMART features that interfered with daily use, or study attrition.

**Conclusions:**

This study is the first to apply contingency management to PrEP adherence. Findings indicated that mSMART is feasible and acceptable. Such an adherence intervention administered via a user-friendly smartphone app can allow for widespread dissemination. Future efficacy trials are needed.

**Trial Registration:**

ClinicalTrials.gov NCT02895893; https://clinicaltrials.gov/ct2/show/NCT02895893 (Accessed by Webcite at http://www.webcitation.org/72JskjDJq)

## Introduction

Pre-exposure prophylaxis (PrEP) in the form of tenofovir disoproxil fumarate and emtricitabine is a highly effective tool to prevent HIV infection [1-9]. However, adherence rates to this once daily medication are highly variable in clinical trials, ranging from 12% to 82% [4,10-15]. This is particularly significant for HIV prevention because the effectiveness of oral PrEP is strongly associated with sustained adherence [3,4,16]. Among those receiving PrEP in a 72-week open-label extension trial, HIV incidence significantly dropped from 4.7 infections per 100 person-years if the drug was not detected in blood to 2.3, 0.6, and 0.0 infections per year if blood levels correlated with participants’ taking less than 2 tablets per week, 2-3 tablets per week, and ≥4 tablets per week, respectively [3]. Young men who have sex with men (MSM) are particularly at risk for HIV and could therefore benefit from PrEP. MSM represent just 2% of the US population but account for 67% of all new HIV diagnoses, which is driven in part by increased rates in young MSM [17]. In conjunction with trials indicating that younger participants, including MSM, are less likely to be adequately adherent to PrEP [3,18,19], interventions that target PrEP adherence are needed.

Despite the importance of PrEP adherence, there are few empirically supported interventions targeting adherence. One pilot trial indicated that a cognitive behavioral intervention, including 4-6 face-to-face sessions, improved PrEP adherence among MSM in comparison to a time-matched control intervention [20]. Although such interventions are promising, easily disseminated and wide-reaching interventions that maintain fidelity to rigorous intervention protocols may further enhance efforts to promote PrEP adherence. Smartphones offer such a platform for personalized and flexible interventions to improve health outcomes that can be administered in a uniform and user-friendly format [21]. Smartphones are used by an increasing segment of the US population (eg, 77% owned one in 2016 up from 35% in 2011) [22]—people who carry smartphones generally have them within reach and switched on at all times [23]. However, despite the fact that there are more than 800 medication adherence apps for a range of conditions, only a few have been widely studied [24]. Although some research is beginning to investigate the use of daily texting to support PrEP adherence [25], a smartphone app targeting HIV prevention that includes PrEP screening [26], and a smartphone app that incorporates PrEP adherence among MSM in an ongoing trial [27], to our knowledge, there are no published studies on medication adherence smartphone apps for PrEP.

Contingency management, administered via smartphones, may be a promising intervention approach for improving PrEP adherence. Contingency management is a behavioral intervention that uses systematic reinforcement dependent on the occurrence of a specific behavior and is effective in improving adherence to medications for a range of medical and psychiatric conditions [28]. Contingency management has been used to successfully improve adherence to antiretroviral medications among HIV-positive and HIV-exposed individuals [29,30], but it has yet to be examined as an intervention for PrEP adherence.

The aim of this study was to develop and pilot test a smartphone-based contingency management intervention, known as mSMART, that targets PrEP adherence. In addition to contingency management, mSMART provides medication reminders, PrEP education, individualized behavioral strategies to address PrEP adherence barriers, and medication adherence feedback. mSMART also assesses adherence in real time using a camera-based medication event-monitoring tool. This was a 4-week open-label, phase I trial. We examined the feasibility and acceptability of mSMART in a sample of young MSM prescribed PrEP in a community setting.

## Methods

### Participants

Inclusion criteria were male at birth, age 18-30 years, self-report having sex with men in the last 6 months, self-report being currently prescribed and taking PrEP for HIV prevention, English speaking, and own an Android or an iPhone compatible with the mSMART smartphone app. Exclusion criteria included significant medical or psychiatric conditions that may interfere with study participation (eg, suicidality) or being unable to attend both study visits. There were no inclusion or exclusion criteria pertaining to the amount of time participants were prescribed PrEP prior to enrollment. Participants were recruited via community advertisements and word of mouth.

**Figure 1:**
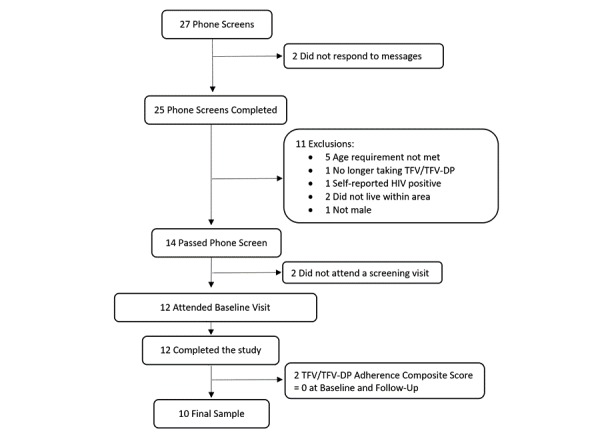
Sample recruitment and participation flowchart. TFV: tenofovir, TFV-DP: tenofovir-diphosphate.

A total of 27 screens conducted over the phone were held, and 14 individuals were invited for the baseline assessment. Individuals were not invited for a baseline visit for the following reasons: they did not respond to phone messages (n=2), they did not meet the age inclusion criterion (n=5), they were not currently prescribed PrEP (n=2), they self-reported HIV-positive status (n=1), they did not live close enough to attend laboratory visits (n=2), and they were not male (n=1). Among the 14 invited individuals for a baseline visit, 2 participants did not attend the visit. A total of 12 participants were consented, but 10 participants were included in this analysis; 2 participants had baseline tenofovir (TFV)/TFV-diphosphate (TFV-DP) levels, indicating that they were not taking PrEP, as seen in Figure 1. We decided to exclude these 2 participants from the analysis post hoc because, in contrast to the final sample of 10, we could not verify that these participants were ever prescribed PrEP using our biomarker analysis. Furthermore, there was concern about the validity of the self-report data these participants provided. For example, both participants stated that they were on PrEP for the past 8 and 9 months and self-reported only missing doses approximately 12 and 2 times over those time periods, respectively. This contrasted with the biomarker analysis that indicated that they had no detectable levels of TFV/TFV-DP.

### Procedures and Measures

In total, 2 laboratory visits were required: baseline and follow-up. Participants were provided the mSMART smartphone app on their smartphone and asked to use it daily during the 4-week period between visits.

#### Baseline Visit

After obtaining the informed consent, participant demographic, medication, and medical and psychiatric history information were collected in a paper-and-pencil format to characterize the sample. Additional questionnaires were administered via a Web-based survey tool during the visit to further characterize the sample. The 6-item Risk Behavior Assessment for MSM [31] recommended by the Centers for Disease Control and Prevention [32,33] was administered to assess HIV risk over the past 6 months. The 9-item Patient Health Questionnaire-9 [34] was used to assess depressed mood over the past 2 weeks. Substance use was assessed using the 10-item Drug Abuse Screening Test [35] and the 10-item Alcohol Use Disorders Identification Test [36]. The number of perceived barriers to PrEP adherence was assessed using the 20-item Adherence Starts with Knowledge questionnaire (ASK-20) [37]—the original version of this scale was modified to inquire about PrEP specifically. This measure was used to characterize perceived PrEP adherence barriers at baseline as well as an outcome measure to compare with follow-up visit ratings.

At the conclusion of the baseline visit, the study participants were registered with the mSMART app by the study team on a secure website [38] and the app was downloaded by participants from the appropriate app distribution platform for their operative system (eg, Apple Store, Google Play). Participants received brief instructions from an experimenter on the functions of mSMART. Overall, the baseline visit took approximately 90-120 minutes to complete.

#### Follow-Up Visit

The ASK-20 questionnaire modified for PrEP was readministered. Treatment acceptability ratings were provided by participants based on responses to individual items examining overall satisfaction with mSMART (ie, “What was your overall satisfaction with mSMART?”), mSMART usability on a daily basis (ie, “How usable was mSMART on a daily basis?”), difficulty learning how to use mSMART (ie, “How difficult was it to learn how to use mSMART?”), willingness to recommend mSMART to others (ie, “Would you recommend mSMART to a friend who is taking a medication?”), and overall user-friendliness of mSMART (ie, “How user-friendly was mSMART?”) on a Likert scale ranging from 1 (not at all) to 4 (extremely). These items were administered in an in-person interview format and were adapted from our past use of a similar scale [39]. The System Usability Scale (SUS) [40] was administered via a Web-based survey tool as a measure of treatment acceptability. SUS is a 10-item scale that assesses responses on a 5-point Likert scale with scores ranging from 0 to 100. Semistructured exit interviews were also conducted for qualitative analysis of participant experiences and perceptions of mSMART. Interview questions addressed topics such as mSMART design features, navigation, barriers to use, and features that facilitated regular use similar to other studies examining participant experience with smartphone-based interventions (eg, [41]).

Participants also completed predetermined tasks within the smartphone app during the follow-up study visit following guidelines from another smartphone app development study [41]. An experimenter sat next to the participant, provided instructions on 6 different tasks, and recorded the time to complete each task. These 6 tasks involved (a) taking a picture of their medication, (b) changing the reminder time for daily dosing, (c) checking how much money was earned using the smartphone app, (d) checking for any questions prompted by mSMART, (e) looking up a detail about medication side effects, and (f) looking up a second detail about medication side effects. The time recorded for each task was based on the first attempt to complete it.

### Bioanalytical Adherence Assessment

Blood samples were collected at both the baseline and follow-up visits to assess for biomarkers of PrEP adherence. The blood samples were used to assess the concentrations of TFV in plasma and intracellular TFV-DP in the upper layer packed cells both to characterize baseline levels and as a comparison with the follow-up assessment levels using methods previously described by Adams et al [42]. These levels were used to develop a semiordinal composite adherence score over the past 4 weeks, ranging from 0 (low or no doses of drug identified: no detectable TFV and <10,000 fmol/mL TFV-DP) to 5 (good adherence: >10 ng/mL TFV and >1,000,000 fmol/mL TFV-DP) [15]. A score of 4 (ie, 4-5 doses per week) or 5 (approximately daily dosing) is typically considered as the level of adherence at which PrEP is efficacious among MSM [43].

#### mSMART Intervention

mSMART was developed through a multistage process initially as a smartphone app for medication adherence for cigarette smokers during quit attempts [44]. It was adapted for PrEP by the research team for this study. The adaptation of mSMART for PrEP was informed by studies on adherence barriers in PrEP trials (eg, [45-48]) and feedback from experts working with and developing interventions for individuals at risk for HIV. The Information, Motivation, and Behavioral Skills (IMB) model [49], which conceptualizes health behavior change as a product of mediators, including information about the behavior, motivation to change, and behavioral skills, guided the refinement of mSMART for PrEP. For example, Information (the first IMB model component) was conveyed through an interactive daily question-and-answer format involving PrEP and HIV knowledge as well as self-assessments of general medication adherence difficulties (see the SMART Desk feature described below). Information was also provided about adherence with mSMART via visual feedback about logging doses each day (see the Treatment Progress feature described below). Motivation (the second IMB model component) to adhere to PrEP was provided in the form of contingent reinforcement when doses were logged daily and daily medication reminders (see the Medication Aide feature described below).

Behavioral Skills (the third IMB component) were taught by mSMART with behavioral skill instruction on how to improve adherence (eg, how to remember to take a daily dose if forgetfulness is a barrier to adherence) and how to cope with short-term side effects that may deter daily adherence (see the Adherence Strategies and Coping Strategies features described below). The IMB model has been used to guide the development of numerous HIV prevention interventions and encapsulates other theoretical HIV risk reduction models (eg, [50]).

**Figure 2:**
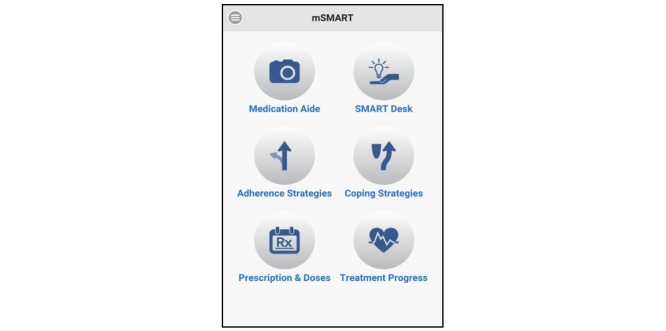
mSMART home screen.

mSMART was used by participants over a 4-week period between the baseline and follow-up visits. mSMART is composed of 6 different components, as seen in Figure 2, that target medication adherence.

Table 1 summarizes the 6 different components of mSMART. The Medication Aide component of mSMART included the contingency management procedures. Upon receiving their daily PrEP reminder notification, participants touched the Medication Aide icon and were then directed to enter a dose of PrEP that they were either about to take or had already taken. In either case, as long as participants reported taking their daily dose of PrEP within 2 hours of their predetermined dosing time each day (dosing times were selected by participants), they received reinforcement. A fixed-ratio schedule of reinforcement was adopted. Participants received feedback that they earned US $2 every time they logged a dose (whether using the camera feature or manually) within a 2-hour window of their daily dosing. Feedback about money earned upon taking their daily dose was provided immediately by mSMART. Over the 4-week period, participants could earn up to US $56. Participants could opt to receive the money that they earned in accordance with the contingency management procedures weekly or at the end of the 4-week intervention period.

#### Data Analysis

##### Adherence Outcomes

Perceived and objective PrEP adherence outcomes were assessed via change scores on ASK-20 and medication adherence scores based on TFV/TFV-DP, respectively.

**Table 1:** Description of mSMART features.

mSMART feature	Description
Medication Aide	Participants used this feature to enter a dose of PrEP^a^ that they were either about to take or had already taken. If a participant indicated that he was about to take his daily PrEP dose, the camera-based medication event-monitoring tool was activated. This involved taking the participant to another screen that prompted him to touch a pill icon that would open up the camera feature on his phone. The mSMART app would automatically take a picture and would take approximately 5-10 s to focus on the pill the participant was holding in his hand—a feedback bar indicating progress was provided over the top portion of the picture. For this study, these pictures were not examined by the study team or saved, although mSMART has that capability. If participants had already taken their daily dose of PrEP, they would manually enter when they took their daily dose of PrEP.
SMART Desk	This component was an interactive space where mSMART prompted brief daily surveys (ie, 1-4 questions per day pertaining to knowledge or concerns about PrEP, knowledge about HIV, and general medication use concerns or problems). These questions were phased out after any 7-day window only if participants were achieving 100% adherence by logging daily PrEP doses in that window, but were resumed if a dose was not logged. For participants who were not logging all daily PrEP doses, they continued to receive daily questions from the SMART Desk. Notifications informing participants of missing a PrEP dose were also provided through the SMART Desk.
Adherence Strategies^b^	This component described behavioral strategies to address PrEP adherence barriers identified in the literature [45-48]. These strategies were prioritized in list form based on responses from participants in the SMART Desk and could be accessed at any time by clicking on the Adherence Strategies icon. For example, if a participant indicated he did not have difficulty remembering to take daily PrEP doses but had a relatively poor understanding of how PrEP prevents HIV on previous SMART Desk questions, the Adherence Strategies component presented educational information about how PrEP prevents HIV before presenting behavioral strategies to help the participant remember to take his medication. Thus, adherence strategies were individualized based on participant responses in the SMART Desk.
Coping Strategies	This feature listed common PrEP side effects. Participants could access a list of side effects at any time and click on any to view strategies to mitigate them. The most common side effects reported in the literature (eg, upset stomach, headache, and vomiting [32,33]) were included.
Prescription and Doses	With this feature, the participants were able to set up their preferred time to receive medication reminders. Participants could change this setting at any time and therefore could modify it on days they anticipated taking PrEP at a different time.
Treatment Progress	This feature provided feedback about the participant’s overall PrEP adherence in the form of percentage of days they logged a dose (within the 2-h window) within the Medication Aide feature. Participants could also click on this feature to see how much money they had earned based on the contingency management procedures.

^a^PrEP: Pre-exposure prophylaxis.

^b^Other examples of adherence strategies that this mSMART component addresses include strategies to organize materials to take medication daily, ways to remember to take medication, education about different aspects of PrEP (eg, explaining why daily adherence is important, describing a typical medical visit schedule once on PrEP, and addressing concerns regarding possible long-term health effects of PrEP), financial aspects related to being on PrEP, information about communicating with health care workers about PrEP and sexual behavior, and eliciting support from family and friends to support PrEP adherence. In addition to accessing adherence strategies by clicking on the Adherence Strategies icon, participants were automatically routed to specific Adherence Strategies from the SMART Desk after completing the questions in the SMART Desk. For example, if the SMART Desk asked about remembering to take medication, the participant would be routed to a strategy within Adherence Strategies to address medication forgetfulness. This routing occurred regardless of the response selected with the intent to increase exposure to a variety of adherence strategies, which was balanced against personalized presentation of strategies based on SMART Desk responses described above.

##### Treatment Feasibility

Feasibility was assessed in the following ways: study attrition rate and any smartphone-mSMART compatibility incidents, daily engagement with mSMART, time needed to complete the predetermined tasks on mSMART, and number of prompts (initiated by either the participant or the experimenter) to assist participants in completing the tasks.

##### Treatment Acceptability

Acceptability was assessed in multiple ways. First, responses to individual treatment acceptability questions about mSMART (ie, overall satisfaction, usability on a daily basis, difficulty learning mSMART, willingness to recommend mSMART to others, and overall user-friendliness) were descriptively analyzed. Second, SUS scores at or above 68 were considered as acceptable [51]. Third, we considered responses to semistructured exit interviews for qualitative analysis. Interviews were digitally recorded, transcribed, and qualitatively analyzed. Qualitative analysis involved identification of categories that emerged. These categories were identified through an iterative process following procedures similar to those involved in our past qualitative approaches [52,53]. That is, an initial list of anticipated categories based on the study team’s experience with mSMART in other populations and separate experiences with young MSM. These categories were subsequently refined based on one of the authors’ experience conducting the exit interviews and reading all interview transcripts. Another rater then read through the transcripts to comment on the category descriptions and identify any additional categories not previously considered. Next, both raters identified any discrepancies in category identification, reconciled these discrepancies, and finalized the categories. Following this process, the 2 raters separately read through the transcripts (n=5 per rater) in a Microsoft Word document and identified category endorsements for each participant. Each interview excerpt that was identified with a category endorsement was transferred to an Excel document so that frequency counts for particular categories could be summed across the full sample. We have adopted similar procedures in past studies [52]. Interrater reliability between raters was assessed on a subset of interview excerpts. Kappa coefficient between raters was .90 when determining whether a category should be endorsed.

## Results

### Sample Characteristics

The sample (N=10) contained predominantly white (n=7) and highly educated (n=7) participants, earning at least an undergraduate degree. The average number of months on PrEP was 8.3 with use ranging from 0.5 to 12 months. All but 1 participant reported being on PrEP for at least 5 months. Each participant exceeded the MSM Risk Index Score of 10 used to evaluate appropriateness for PrEP [32,33], indicating high risk for HIV. In addition, participants yielded low scores for depressed mood, drug use, and alcohol use (see Table 2 for a summary).

### Adherence Outcomes

#### Objective Adherence

PrEP composite adherence scores based on TFV/TFV-DP values indicated that PrEP adherence increased for 30% (3/10) of the sample and did not change for 70% (7/10) of the sample. For participants who did not indicate any change, PrEP adherence scores were already at a level considered efficacious (ie, ≥4 doses per week) at baseline. Among the 3 participants whose PrEP adherence scores increased, 1 had a baseline score below what is considered efficacious. No PrEP composite adherence scores decreased. Table 3 provides the baseline and follow-up scores.

#### Perceived Adherence

The perceived number of barriers to PrEP adherence was measured using the modified ASK-20 at baseline and follow-up. A comparison of scores within participants indicated an increase in the number of perceived barriers for 1 participant. This participant indicated on an ASK-20 item that his belief that PrEP was helpful in reaching his overall health goals had decreased. However, 3 participants indicated that the number of barriers they perceived to PrEP adherence decreased, including barriers associated with the financial cost of PrEP. There was no change in modified ASK-20 scores for 50% (5/10) of the sample. One participant did not complete the modified ASK-20 at follow-up.

### Treatment Feasibility

**Figure 3:**
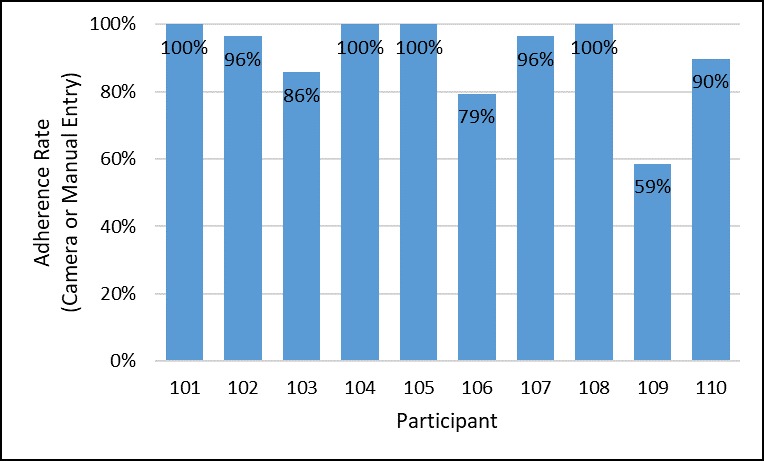
Percentage of time the individual participants logged a dose in mSMART using either the camera-based medication event-monitoring tool or manual entry option.

**Figure 4:**
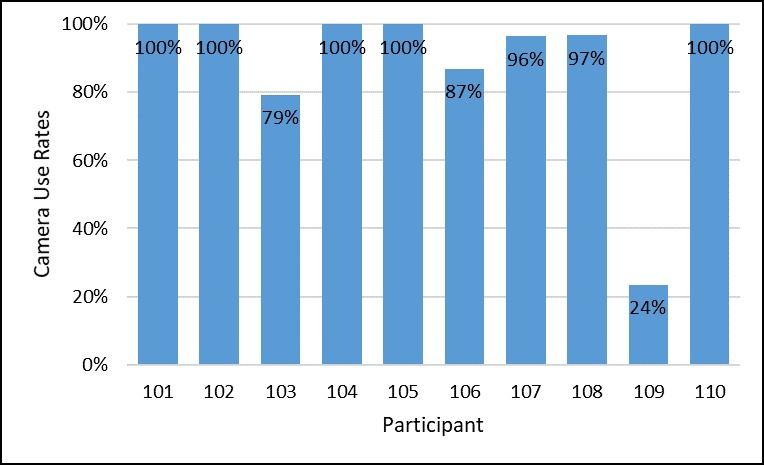
Percentage of time the camera-based medication event-monitoring tool was used among participants when they logged a dose in mSMART.

There was no study attrition. Furthermore, there were no smartphone-mSMART incompatibility events in which the mSMART app was not able to function on a study participant’s phone. In terms of daily engagement with mSMART, participants logged a PrEP dose in mSMART (using either the camera-based medication event-monitoring tool or manual entry option) 91% of the time over the 4-week intervention period, as seen in Figure 3, with the mean amount earned per contingency management guidelines being US $53 per participant. Among these logged doses in mSMART, 88% involved the use of the camera-based medication event-monitoring tool, as seen in Figure 4. Overall, 40% (4/10) of the sample did not miss any days logging a PrEP dose in mSMART. An additional 40% (4/10) did not log a PrEP dose in mSMART between 1 and 5 days while in the study. Among the remaining participants, 1 did not log a PrEP dose for 6 days and the other did not log a PrEP dose for 12 days. Furthermore, 70% (7/10) of the sample responded to all of the mSMART daily surveys. During the follow-up study visit, all participants were able to complete each of the 6 predetermined tasks on mSMART without any prompts (initiated by either the participant or the experimenter). The amount of time it took to complete these tasks was 5.39 seconds (average across all tasks; see Multimedia Appendix 1).

**Table 2:** Sample characteristics (N=10).

Characteristic		Value
Age, mean (SD)		24.10 (2.38)
**Race, n (%)**		
	Black	0 (0)
	White	7 (70)
	Asian	2 (20)
	Multiracial	1 (10)
**Ethnicity, n (%)**		
	Hispanic	0 (0)
	Not Hispanic	9 (90)
	Not reported	1 (10)
**Education, n (%)**		
	High school graduate	1 (10)
	Partial college	2 (20)
	College graduate	5 (50)
	Postgraduate studies	2 (20)
**Employment status, n (%)**		
	Full-time	3 (30)
	Part-time	2 (20)
	Assistance	0 (0)
	Unemployed	1 (10)
	Dependent or student	3 (30)
	Not reported	1 (10)
**Salary range, n (%)**		
	US $0-$10,000	3 (30)
	US $10,000-$25,000	3 (30)
	US $25,000-$50,000	0 (0)
	US $50,000-$75,000	2 (20)
	>US $75,000	1 (10)
	Not reported	1 (10)
Months prescribed PrEP^a^, mean (SD)		8.30 (3.45)
MSM^b^ Risk Index Score^c^, mean (SD)		21.50 (5.48)
**Smartphone, n (%)**		
	iPhone	9 (90)
	Android	1 (10)
**Patient Health Questionnaire-9, n (%)**		
	Minimal depression (scores=0-5)	9 (90)
	Mild depression (score=6)	1 (10)
**Drug Abuse Screening Test, n (%)**		
	None	7 (70)
	Low	3 (30)
**Alcohol Use Disorders Identification Test, n (%)**		
	Low risk	10 (100)

^a^PrEP: Pre-exposure prophylaxis.

^b^MSM: Men who have sex with men.

^c^100% of the sample exceeded the cut-off score of 10 and therefore are recommended to evaluate for PrEP per Centers for Disease Control and Prevention guidelines [32,33].

**Table 3:** Frequency of pre-exposure prophylaxis (PrEP) composite adherence scores at study baseline and follow-up visits.

Composite score^a^	Baseline (%)	Follow-up (%)
0	0 (0)	0 (0)
1	1 (10)	0 (0)
2	0 (0)	0 (0)
3	0 (0)	0 (0)
4	8 (80)	7 (70)
5	1 (10)	3 (30)

^a^Composite scores were based on concentrations of tenofovir (TFV) in plasma and intracellular TFV-diphosphate (TFV-DP) in upper layer packed cells. Scores assess adherence in the past 4 weeks, ranging from 0 (low or no doses of drug identified) to 5 (good adherence). A score of 4 (ie, 4-5 doses per week) or 5 (approximately daily dosing) is typically considered as a good level of adherence in which PrEP is efficacious. Because 1 participant was on PrEP for only 2 weeks, the baseline visit adherence score for this participant could have been artificially lower as a result of taking PrEP for a shorter duration in comparison to other study participants (ie, all other participants reported being on it for at least 5 months). However, this participant yielded a baseline adherence score of 4, indicating an adequate level of protection since starting on PrEP and that his score was likely not artificially lower.

### Treatment Acceptance: Quantitative Analysis

In-person interview items at the follow-up visit indicated that the mean rating on a scale of 1 (not at all) to 4 (extremely) for overall satisfaction with mSMART was 2.80 (SD 0.63), mSMART usability on a daily basis was 3.50 (SD 0.53), willingness to recommend mSMART to others prescribed PrEP was 2.70 (SD 0.82), and user-friendliness of mSMART was 2.80 (SD 0.79). Participants also indicated that difficulty learning how to use mSMART was 1.20 (SD 0.42).

On the SUS, with total scores ranging from 0 to 100, the mean score was 68.25 (SD 15.10). Using a score of ≥68 as an indicator of mSMART user-acceptability, 60% (6/10) of the sample met this cut-off.

### Treatment Acceptance: Qualitative Analysis

We classified participant comments about mSMART into the following 6 different categories: (1) mSMART features that were liked or disliked, (2) daily mSMART use, (3) mSMART aesthetics, (4) learning how to use mSMART, (5) mSMART features that should be added, and (6) the likelihood of using mSMART. The first 4 domains were further subdivided into comments that were either positive or negative feedback about mSMART (see Multimedia Appendix 2 for a summary of endorsement rates across domains).

#### mSMART Features: Liked and Disliked

The majority of the sample, 8 participants, commented on features that they both liked and disliked, and the other 2 participants commented only on features that they liked and did not report any dislikes. Particular mSMART features that were most frequently mentioned included using the camera feature (5 participants liked, 2 participants disliked, 2 participants both liked and disliked, and 1 participant did not comment on this feature), receiving daily questions (5 participants liked, 0 participants disliked, 3 participants both liked and disliked, and 2 participants did not comment on this feature), and receiving medication reminders (5 participants liked, 1 participant disliked, 1 participant both liked and disliked, and 3 participants did not comment on this feature), for example, when asked about his overall impression of mSMART, 1 participant responded in the following way about the medication reminders:

I think [mSMART] is helpful, I mean, because it also offers some useful information on Truvada and, uhh, the most important thing is it offers reminders and I mean, at least for now, I still need the reminder to remind me to take my medication. Before I started using this app...it’s really easy to forget every day.

Another participant commented on how the daily questions from the SMART Desk were helpful as follows:

[mSMART] figured out what I struggled with by the questions.

Although some participants indicated that they both liked and disliked the camera feature, most of the comments about disliking the camera feature actually involved initial difficulty learning how to use this feature, for example, 1 participant stated the following:

I think there were like, especially in the first week, there were four or five times I would try to take a picture of the pill and it went straight to uhhh, it wouldn’t, it didn’t read it. And mainly it was times when I was taking the picture and it was too dark, right? I was just like in my room in the morning and didn’t have any lights on, or in our kitchen and it was just really dark. And that was frustrating. Umm, it wasn’t so, I mean it was a minor inconvenience in the grand scheme of the world, right?

After describing this initial experience with the camera feature, this participant went on to say,

Personally, I definitely think that taking a photo was good.

Other participants indicated that the camera feature helped with consolidating memories to increase confidence that they took their medication, for example,

...using the camera like, really forced me to use it and kind of was a mental check guard for myself to make sure I took the medication...Like I was telling you that, I’m like, did I take the pill? Was it yesterday when I was going to work? Or was that today? And so [inaudible] taking the picture at 5:37, like I did do it today. So I’m not confused. I know when I took it, and that was today.

The money earned in accordance with the contingency management procedures was not among the mSMART features that participants considered to find helpful, that is, only 4 participants indicated liking the contingency management payment feature of mSMART; 2 participants said that it was not a helpful feature in adhering to PrEP and another 4 participants indicated that they neither liked nor disliked this feature.

#### Daily Use: Facilitators and Barriers

Four participants commented on factors that facilitated successful daily use of mSMART, whereas 6 participants commented on factors that both facilitated mSMART use and barriers to mSMART use. The most frequently endorsed factor facilitating daily mSMART use (7 participants) was using it at the time they received a medication reminder. For example, 1 participant stated the following:

I pretty much only used it when I needed to log my, umm, medication, which was at night.

Regarding barriers to daily use, the most frequently identified feature was that the speed of mSMART was too slow (ie, time to transition from one screen to the next or to perform a function) and was therefore a barrier to use (2 participants):

So, sometimes when you click on the medic—, like the camera function, it takes a second and then it’ll go, umm, and then it’ll take a minute to get to the next slide and the next screen or whatever you want to call it, which is fine, but I’m just saying like for someone who is going to use it every day and does not have the incentive of here’s...money at the end of the trial, you know what I mean? It could be, people could, someone might get frustrated.

#### mSMART Aesthetics: Liked and Disliked

An equal proportion of participants either commented that they disliked mSMART aesthetics (4 participants) or both liked and disliked the aesthetics of the app (4 participants); the other 2 participants commented either only on aesthetics that they liked (n=1) or did not comment about aesthetics at all (n=1). The most frequently identified aesthetic that was disliked was how the content was displayed in text format (7 participants), for example,

I would go to some of the coping strategies just to like look through them, and I would say, like, you open it, and there’s kind of just a large block of text—that might be a little intimidating....on the one hand I thought it was helpful because it felt like a pretty clinical tone, umm, like from a healthcare provider, like in a good way. Like, if that’s what you want, you know, if you want that...But, then sometimes I was thinking that maybe I would want a more just like a friend...

The most frequently identified aesthetics that was liked was the overall design of mSMART (4 participants), for example,

I thought it was pretty well-designed. And I guess, yeah, I mean it was clearly laid out to me. Umm, the, like the functions, like everything opened when you tapped it. There was no like glitch, there was no, the camera, everything worked.

Similarly, another participant stated the following about the design when asked:

I like the simple breakdown into the six different sections, I think that’s what makes it user-friendly. Umm, I mean, it’s easy to follow when you go into like the different coping strategies and whenever you try to highlight the hyperlinks are really easy to kind of delineate what you’re looking for.

#### Learning How to Use mSMART: Easy and Difficult

Although 9 participants indicated that learning how to use mSMART was easy, 1 participant indicated that it was difficult. Typical comments about learning how to use mSMART included

I think the app is actually pretty, uhh, like user-friendly. It doesn’t, it doesn’t take a whole lot of learning.

#### Features of mSMART That Should Be Added

In total, 90% (9/10) of participants commented on features of mSMART that they thought should be modified. The most commonly endorsed feature that should be added was a snooze option for medication reminders (4 participants), for example, when discussing modifying the alarm feature of mSMART, 1 participant suggested the following change:

Almost like on your phone if you snoozed or something...If something happens, it will alert you again....to physically turn it off almost.

#### Likelihood of Using mSMART

The majority of the sample, 9 participants, commented on how they thought mSMART would be most appropriate for individuals either just starting PrEP or those who have PrEP adherence problems. In particular, 6 participants—all of whom reported taking PrEP for at least 5 months—commented on how it would have been helpful to use mSMART when they began taking PrEP. For example, 1 participant commented the following on how mSMART would have been helpful when starting on PrEP:

I do think, like the first two months probably would’ve been the time this app would’ve been the most helpful...Cause there were also times that I straight up, like I forgot if I had taken it or not; in that first month.

Another participant spoke less about his own initial difficulties with PrEP adherence but spoke more broadly about individuals initiating PrEP as follows:

...as more and more people learn about it and find out about it that information might be less, so that there might be more questions about understanding side effects, especially in the first month where you’re most likely to have side effects. So like, I think that [is] where it can be useful. So like figuring out how do I cope with these side effects? Are they going to go away? What’s the duration?...Umm I think, one thing I can imagine is like, you know, suppose that when you’re first starting your medication, you’re less likely to have a routine, so you’re more likely to miss a dose, and in some cases you might wonder, well like what, let’s say I usually take my dose at 8 in the morning and its 3 in the afternoon, and I just realized I didn’t take my pill, should I take my pill or not? That’s a question that I think people might have, and your doctor may or may not have given you guidance on what to do in that situation...So, that’s an area where I can imagine the role this app can fill.

## Discussion

The large-scale implementation of PrEP is an ongoing challenge that requires diverse models of delivery addressing multiple facets of the PrEP continuum of care [54,55]. This study was a 4-week pilot trial of mSMART as a mobile health PrEP adherence intervention. Adherence outcomes, treatment feasibility, and treatment acceptability were examined in 10 young MSM already prescribed PrEP in the community. Findings from this treatment development study are preliminary but yield promising results and indications for treatment refinement for future efficacy trials.

PrEP adherence outcomes were measured both objectively and subjectively. For the former, PrEP composite adherence scores based on TFV/TFV-DP were examined. Although baseline scores indicated a high level of adherence prior to using mSMART with 90% (9/10) of the sample was at or above a level of PrEP adherence considered as efficacious and therefore a ceiling effect would likely occur, 30% (3/10) of the sample’s scores improved at follow-up. In addition, no composite adherence scores worsened over the course of the study. Our use of biomarkers as an adherence outcome is a strength given that there is substantial within-subject variability in adherence based on the measurement method selected among individuals on PrEP [56]. Alternative methods such as self-report [57] and electronic pill bottles [58] among young MSM have noted limitations. In terms of a future direction, because PrEP adherence scores showed an already high rate at baseline, future studies are needed that would address whether mSMART improves PrEP adherence among those with a poor medication adherence history and whether this impact on adherence is clinically significant.

The subjective measure of adherence was an adapted medication questionnaire measuring perceived PrEP adherence barriers administered at baseline and follow-up. Although 50% (5/10) of the sample did not report any change in barriers to PrEP adherence, 30% (3/10) reported a decrease in barriers and 10% (1/10) reported an increase in barriers. Participants who reported a decrease in barriers indicated that factors such as barriers associated with the financial cost of PrEP; the participant who reported an increase in barriers indicated that the belief that PrEP was helpful in reaching overall health goals had changed. Although these changes in perceived barriers (either increasing or decreasing) emerged, future studies that include a control condition are needed to address whether these changes occurred because of mSMART or other factors. Overall, across the methods of adherence examined in this study, the findings were relatively consistent.

mSMART feasibility was positive as evidenced by 0% (0/10) study attrition, the absence of any smartphone incompatibility events, and daily engagement with mSMART. Regarding daily engagement, we looked at the rates of logged PrEP doses and the proportion that responded to all of the mSMART daily surveys. Using either the camera-based medication event-monitoring tool or the manual entry option within a 2-hour window of when participants identified the time they should take their PrEP pill, the overall adherence rate was 91%. Although contingency management guidelines in this trial considered a medication event as valid either if there was a picture taken of the PrEP pill or if it was entered retrospectively, a more methodologically rigorous contingency management approach would require an objective assessment of behavior that does not rely on self-report (eg, use of the camera-based medication event-monitoring tool only). Given that the majority of times medication adherence was reported via mSMART involved camera-based entries (88%), a more rigorous contingency management intervention appears feasible in future mSMART studies. In terms of responses to daily surveys on mSMART, 70% (7/10) of participants responded to all of the questions.

Feasibility was also examined by measuring the time it took participants to complete different tasks on mSMART. Although there is no standardization of scores on these tasks (ie, the number of seconds to complete each task within mSMART), the performance on these tasks can inform treatment development efforts, such as determining whether basic procedures within the smartphone app are understood and can be executed independently [41]. In this sample, no prompts were requested by participants and the majority of tasks (92%, 55/60 tasks completed across the whole sample) were carried out in 10 seconds or less, which indicated that mSMART was a feasible tool for young MSM on PrEP.

Acceptability of mSMART was examined with mixed results. Ratings on a 4-point Likert scale indicated that participants on average “moderately” agreed that mSMART was usable on a daily basis and somewhat less than “moderately” agreed that overall, they were satisfied with mSMART as an intervention to improve PrEP adherence, they would be willing to recommend mSMART to others on PrEP, and it was user-friendly. Difficulty learning how to use mSMART was minimal. SUS indicated that 60% (6/10) of the sample found the mSMART intervention to be usable. Although a sample size of 10 is small, guidelines for SUS indicate that it measures perceived usability of a system with a small sample around this size [59,60].

Qualitative analyses of exit interviews were conducted to complement the quantitative analyses of acceptability and examined aspects of mSMART that could be maintained, discarded, or adapted in future iterations. Although some features of mSMART were generally perceived favorably (eg, use of reminders, the camera feature, and daily questions), participants indicated that even these features could be adapted in future trials. For example, some participants expressed that a “snooze” function or multiple reminder alarms should be added. One particularly notable feedback theme was that mSMART was too text heavy with suggestions to minimize wording and make the display of such wording more visually appealing (eg, in bulleted formatting, as opposed to paragraphs). Although mSMART is a multicomponent intervention (eg, contingency management, behavioral skills training, and use of reminders), one feature that we anticipated to emerge in our exploratory qualitative analysis was for participants to view contingency management favorably. However, other features of mSMART emerged that were more favored than contingency management. Therefore, as mSMART undergoes further development, comparative trials should consider whether mSMART is viewed just as favorably without contingency management. This aspect of our findings pertain to contingency management’s acceptability and not contingency management’s efficacy. It is unclear whether the magnitude of the reinforcer adopted in this study actually impacted PrEP adherence. In addition, it may be that reinforcer saliency (ie, US $2 for each logged dose) was too low for participants to find engaging and other contingency management approaches may be warranted to improve PrEP acceptability. Although this could come in the form of a higher dollar amount as a reinforcer, the cost of such contingency management approaches may be prohibitive. To reconcile this, studies should consider lower cost contingency management approaches that are engaging (eg, the “fishbowl” technique) [61-63] or a time-limited use of mSMART with higher reinforcer amounts (eg, during PrEP initiation, as was recommended in our qualitative analysis).

Future studies are needed to build on these pilot trial findings. In addition to the factors mentioned above, efficacy trials are needed to examine whether mSMART improves PrEP adherence in comparison to a control group. This would necessitate larger samples that are statistically powered to detect group differences as well as consideration of sample composition (eg, those initiating PrEP or who have struggled with PrEP adherence at baseline, as opposed to the sample in this study in which 90% (9/10) had protective levels at baseline and therefore may have already established adherence habits). Relatedly, although this study examined a group at risk for HIV infection—young MSM—young black MSM are a particularly at-risk group [17]. However, the sample for this study was 70% (7/10) white and did not contain any black MSM, which limits generalizability. Finally, because PrEP use is extending into adolescent MSM, adherence interventions are needed that address unique challenges that emerge in working with this younger age group than those included in this study [18].

In conclusion, this was a phase I trial of a mobile health intervention that aims to improve PrEP adherence. To our knowledge, mSMART is the first PrEP adherence intervention administered via smartphones to integrate contingency management. Given its mobile health format and the ubiquity of smartphone use among younger populations recommended for PrEP [22], this is a PrEP adherence intervention that would be scalable and likely easily disseminated into clinical care settings. In clinical practice, mSMART could be integrated with electronic health records and allow for real-time communication between health care providers and patients. However, although our findings indicate that mSMART is a promising intervention to improve adherence rates, the results are preliminary and future studies are needed to demonstrate efficacy. These studies should also consider our findings indicating areas in which mSMART can be adapted to more comprehensively meet the needs of young MSM prescribed PrEP.

## Appendix

Multimedia Appendix 1 Time it took all participants to complete each of the 6 predetermined tasks on mSMART without any prompts during the follow-up study visit.

Multimedia Appendix 2 Summary of endorsement rates across domains.

## Abbreviations

ASK-20: 20-item Adherence Starts with Knowledge questionnaire
IMB: Information, Motivation, and Behavioral
MSM: Men who have sex with men
PrEP: Pre-exposure prophylaxis
SUS: System Usability Scale
TFV: tenofovir
TFV-DP: tenofovir-diphosphate

## References

[1] Grant R, Anderson P, McMahan V, Liu A, Amico K, Mehrotra M Results of the iPrEx open-label extension (iPrEx OLE) in men and transgender women who have sex with men: PrEP uptake, sexual practices, and HIV incidence.

[2] Molina J, Capitant C, Charreau I, Meyer L, Spire B, Pialoux G On demand PrEP with oral TDF-FTC in MSM: results of the ANRS Ipergay Trial.

[3] Grant RM, Anderson PL, McMahan V, Liu A, Amico KR, Mehrotra M, Hosek S, Mosquera C, Casapia M, Montoya O, Buchbinder S, Veloso VG, Mayer K, Chariyalertsak S, Bekker L, Kallas EG, Schechter M, Guanira J, Bushman L, Burns DN, Rooney JF, Glidden DV, iPrEx ST (2014). Uptake of pre-exposure prophylaxis, sexual practices, and HIV incidence in men and transgender women who have sex with men: a cohort study. Lancet Infect Dis.

[4] Grant RM, Lama JR, Anderson PL, McMahan V, Liu AY, Vargas L, Goicochea P, Casapía M, Guanira-Carranza JV, Ramirez-Cardich ME, Montoya-Herrera O, Fernández T, Veloso VG, Buchbinder SP, Chariyalertsak S, Schechter M, Bekker L, Mayer KH, Kallás EG, Amico KR, Mulligan K, Bushman LR, Hance RJ, Ganoza C, Defechereux P, Postle B, Wang F, McConnell JJ, Zheng J, Lee J, Rooney JF, Jaffe HS, Martinez AI, Burns DN, Glidden DV, iPrEx ST (2010). Preexposure chemoprophylaxis for HIV prevention in men who have sex with men. N Engl J Med.

[5] Baeten J, Heffron R, Kidoguchi L, Celum C Near elimination of HIV transmission in a demonstration project of PrEP and ART.

[6] Martin M, Mock PA, Curlin ME, Vanichseni S Preliminary follow-up of injecting drug users receiving preexposure prophylaxis.

[7] Peterson L, Taylor D, Roddy R, Belai G, Phillips P, Nanda K, Grant R, Clarke EEK, Doh AS, Ridzon R, Jaffe HS, Cates W (2007). Tenofovir disoproxil fumarate for prevention of HIV infection in women: a phase 2, double-blind, randomized, placebo-controlled trial. PLoS Clin Trials.

[8] McCormack S, Dunn D Pragmatic open-label randomised trial of preexposure prophylaxis: the PROUD Study.

[9] Grohskopf LA, Chillag KL, Gvetadze R, Liu AY, Thompson M, Mayer KH, Collins BM, Pathak SR, Oʼhara B, Ackers ML, Rose CE, Grant RM, Paxton LA, Buchbinder SP (2013). Randomized trial of clinical safety of daily oral tenofovir disoproxil fumarate among HIV-uninfected men who have sex with men in the United States. J Acquir Immune Defic Syndr.

[10] Baeten JM, Donnell D, Ndase P, Mugo NR, Campbell JD, Wangisi J, Tappero JW, Bukusi EA, Cohen CR, Katabira E, Ronald A, Tumwesigye E, Were E, Fife KH, Kiarie J, Farquhar C, John-Stewart G, Kakia A, Odoyo J, Mucunguzi A, Nakku-Joloba E, Twesigye R, Ngure K, Apaka C, Tamooh H, Gabona F, Mujugira A, Panteleeff D, Thomas KK, Kidoguchi L, Krows M, Revall J, Morrison S, Haugen H, Emmanuel-Ogier M, Ondrejcek L, Coombs RW, Frenkel L, Hendrix C, Bumpus NN, Bangsberg D, Haberer JE, Stevens WS, Lingappa JR, Celum C, Partners PST (2012). Antiretroviral prophylaxis for HIV prevention in heterosexual men and women. N Engl J Med.

[11] Choopanya K, Martin M, Suntharasamai P, Sangkum U, Mock PA, Leethochawalit M, Chiamwongpaet S, Kitisin P, Natrujirote P, Kittimunkong S, Chuachoowong R, Gvetadze RJ, McNicholl JM, Paxton LA, Curlin ME, Hendrix CW, Vanichseni S, Bangkok TSG (2013). Antiretroviral prophylaxis for HIV infection in injecting drug users in Bangkok, Thailand (the Bangkok Tenofovir Study): a randomised, double-blind, placebo-controlled phase 3 trial. Lancet.

[12] Marrazzo JM, Ramjee G, Richardson BA, Gomez K, Mgodi N, Nair G, Palanee T, Nakabiito C, van DSA, Noguchi L, Hendrix CW, Dai JY, Ganesh S, Mkhize B, Taljaard M, Parikh UM, Piper J, Mâsse B, Grossman C, Rooney J, Schwartz JL, Watts H, Marzinke MA, Hillier SL, McGowan IM, Chirenje ZM (2015). Tenofovir-based preexposure prophylaxis for HIV infection among African women. N Engl J Med.

[13] Thigpen MC, Kebaabetswe PM, Paxton LA, Smith DK, Rose CE, Segolodi TM, Henderson FL, Pathak SR, Soud FA, Chillag KL, Mutanhaurwa R, Chirwa LI, Kasonde M, Abebe D, Buliva E, Gvetadze RJ, Johnson S, Sukalac T, Thomas VT, Hart C, Johnson JA, Malotte CK, Hendrix CW, Brooks JT, TDF2 SG (2012). Antiretroviral preexposure prophylaxis for heterosexual HIV transmission in Botswana. N Engl J Med.

[14] Van Damme L, Corneli A, Ahmed K, Agot K, Lombaard J, Kapiga S, Malahleha M, Owino F, Manongi R, Onyango J, Temu L, Monedi MC, Mak'Oketch P, Makanda M, Reblin I, Makatu SE, Saylor L, Kiernan H, Kirkendale S, Wong C, Grant R, Kashuba A, Nanda K, Mandala J, Fransen K, Deese J, Crucitti T, Mastro TD, Taylor D, FEM-Pr EG (2012). Preexposure prophylaxis for HIV infection among African women. N Engl J Med.

[15] Corneli AL, Deese J, Wang M, Taylor D, Ahmed K, Agot K, Lombaard J, Manongi R, Kapiga S, Kashuba A, Van DL, FEM-Pr EG (2014). FEM-PrEP: adherence patterns and factors associated with adherence to a daily oral study product for pre-exposure prophylaxis. J Acquir Immune Defic Syndr.

[16] Amico KR, Stirratt MJ (2014). Adherence to preexposure prophylaxis: current, emerging, and anticipated bases of evidence. Clin Infect Dis.

[17] Centers for Disease Control and Prevention (2015). https://stacks.cdc.gov/view/cdc/37333. Archived at: http://www.webcitation.org/6y6uQgvT4.

[18] Koss CA, Hosek SG, Bacchetti P, Anderson PL, Liu AY, Horng H, Benet LZ, Kuncze K, Louie A, Saberi P, Wilson CM, Gandhi M (2018). Comparison of measures of adherence to HIV preexposure prophylaxis among adolescent and young men who have sex with men in the United States. Clin Infect Dis.

[19] Hosek SG, Landovitz RJ, Kapogiannis B, Siberry GK, Rudy B, Rutledge B, Liu N, Harris DR, Mulligan K, Zimet G, Mayer KH, Anderson P, Kiser JJ, Lally M, Brothers J, Bojan K, Rooney J, Wilson CM (2017). Safety and feasibility of antiretroviral preexposure prophylaxis for adolescent men who have sex with men Aged 15 to 17 years in the United States. JAMA Pediatr.

[20] Mayer KH, Safren SA, Elsesser SA, Psaros C, Tinsley JP, Marzinke M, Clarke W, Hendrix C, Wade TS, Haberer J, Mimiaga MJ (2017). Optimizing pre-exposure antiretroviral prophylaxis adherence in men who have sex with men: results of a pilot randomized controlled trial of “Life-Steps for PrEP”. AIDS Behav.

[21] Miller G (2012). The smartphone psychology manifesto. Perspect Psychol Sci.

[22] (2017). Pew Research Center.

[23] Klasnja P, Pratt W (2012). Healthcare in the pocket: mapping the space of mobile-phone health interventions. J Biomed Inform.

[24] Ahmed I, Ahmad NS, Ali S, Ali S, George A, Saleem DH, Uppal E, Soo J, Mobasheri MH, King D, Cox B, Darzi A (2018). Medication adherence apps: review and content analysis. JMIR Mhealth Uhealth.

[25] Moore DJ, Jain S, Dubé MP, Daar ES, Sun X, Young J, Corado K, Ellorin E, Milam J, Collins D, Blumenthal J, Best BM, Anderson P, Haubrich R, Morris SR (2017). Randomized controlled trial of daily text messages to support adherence to PrEP in at-risk for HIV individuals: the TAPIR Study. Clin Infect Dis.

[26] Sullivan PS, Driggers R, Stekler JD, Siegler A, Goldenberg T, McDougal SJ, Caucutt J, Jones J, Stephenson R (2017). Usability and acceptability of a mobile comprehensive HIV prevention app for men who have sex with men: a pilot study. JMIR Mhealth Uhealth.

[27] Available from: https://clinicaltrials.gov/ct2/show/NCT03320512?term=hightow-weidman&rank=3. Archived at: http://www.webcitation.org/6y73X0EQd.

[28] Petry NM, Rash CJ, Byrne S, Ashraf S, White WB (2012). Financial reinforcers for improving medication adherence: findings from a meta-analysis. Am J Med.

[29] Mbuagbaw L, Sivaramalingam B, Navarro T, Hobson N, Keepanasseril A, Wilczynski NJ, Haynes RB, Patient Adherence Review (PAR) Team (2015). Interventions for enhancing adherence to antiretroviral therapy (ART): a systematic review of high quality studies. AIDS Patient Care STDS.

[30] Landovitz RJ, Fletcher JB, Shoptaw S, Reback CJ (2015). Contingency management facilitates the use of postexposure prophylaxis among stimulant-using men who have sex with men. Open Forum Infect Dis.

[31] Menza TW, Hughes JP, Celum CL, Golden MR (2009). Prediction of HIV acquisition among men who have sex with men. Sex Transm Dis.

[32] (2014). United States Public Health Service.

[33] United States Public Health Service Preexposure prophylaxis for the prevention of HIV infection in the United States: clinical providers supplement.

[34] Kroenke K, Spitzer RL, Williams JB (2001). The PHQ-9: validity of a brief depression severity measure. J Gen Intern Med.

[35] Skinner HA (1982). The drug abuse screening test. Addict Behav.

[36] Saunders JB, Aasland OG, Babor TF, de LFJR, Grant M (1993). Development of the Alcohol Use Disorders Identification Test (AUDIT): WHO collaborative project on early detection of persons with harmful alcohol consumption--II. Addiction.

[37] Hahn SR, Park J, Skinner EP, Yu-Isenberg KS, Weaver MB, Crawford B, Flowers PW (2008). Development of the ASK-20 adherence barrier survey. Curr Med Res Opin.

[38] Intelligent Automation, Inc.

[39] Mitchell JT, McIntyre EM, English JS, Dennis MF, Beckham JC, Kollins SH (2017). A pilot trial of mindfulness meditation training for ADHD in adulthood: impact on core symptoms, executive functioning, and emotion dysregulation. J Atten Disord.

[40] Brooke J (1996). SUS-A quick and dirty usability scale. Usability Eval Ind.

[41] Vilardaga R, Rizo J, Kientz JA, McDonell MG, Ries RK, Sobel K (2016). User Experience evaluation of a smoking cessation app in people with serious mental illness. Nicotine Tob Res.

[42] Adams JL, Sykes C, Menezes P, Prince HMA, Patterson KB, Fransen K, Crucitti T, De BI, Van DL, Kashuba ADM (2013). Tenofovir diphosphate and emtricitabine triphosphate concentrations in blood cells compared with isolated peripheral blood mononuclear cells: a new measure of antiretroviral adherence?. J Acquir Immune Defic Syndr.

[43] Anderson PL, Glidden DV, Liu A, Buchbinder S, Lama JR, Guanira JV, McMahan V, Bushman LR, Casapía M, Montoya-Herrera O, Veloso VG, Mayer KH, Chariyalertsak S, Schechter M, Bekker L, Kallás EG, Grant RM, iPrEx ST (2012). Emtricitabine-tenofovir concentrations and pre-exposure prophylaxis efficacy in men who have sex with men. Sci Transl Med.

[44] Nanda N, Satapathy G, Holt L, McClernon FJ, Mitchell JT mSMART: personalized mobile app for improving medication adherence.

[45] Amico KR, Mansoor LE, Corneli A, Torjesen K, van der Straten A (2013). Adherence support approaches in biomedical HIV prevention trials: experiences, insights and future directions from four multisite prevention trials. AIDS Behav.

[46] Gilmore HJ, Liu A, Koester KA, Amico KR, McMahan V, Goicochea P, Vargas L, Lubensky D, Buchbinder S, Grant R (2013). Participant experiences and facilitators and barriers to pill use among men who have sex with men in the iPrEx pre-exposure prophylaxis trial in San Francisco. AIDS Patient Care STDS.

[47] Haberer JE, Bangsberg DR, Baeten JM, Curran K, Koechlin F, Amico KR, Anderson P, Mugo N, Venter F, Goicochea P, Caceres C, O'Reilly K (2015). Defining success with HIV pre-exposure prophylaxis: a prevention-effective adherence paradigm. AIDS.

[48] Liu AY, Cohen SE, Vittinghoff E, Anderson PL, Doblecki-Lewis S, Bacon O, Chege W, Postle BS, Matheson T, Amico KR, Liegler T, Rawlings MK, Trainor N, Blue RW, Estrada Y, Coleman ME, Cardenas G, Feaster DJ, Grant R, Philip SS, Elion R, Buchbinder S, Kolber MA (2016). Preexposure prophylaxis for HIV infection integrated with municipal- and community-based sexual health services. JAMA Intern Med.

[49] Fisher J, Fisher W (2000). Theoretical approaches to indivdual-level change in HIV risk behavior. Peterson JL, DiClemente RJ. editors. Handbook of HIV prevention. 1st ed.

[50] Aliabadi N, Carballo-Dieguez A, Bakken S, Rojas M, Brown W, Carry M, Mosley JP, Gelaude D, Schnall R (2015). Using the information-motivation-behavioral skills model to guide the development of an HIV prevention smartphone application for high-risk MSM. AIDS Educ Prev.

[51] Sauro J (2011). A practical guide to the system usability scale: background, benchmarks,best practices. Measuring Usability LLC.

[52] Mitchell JT, Sweitzer MM, Tunno AM, Kollins SH, McClernon FJ (2016). “I use weed for my ADHD”: a qualitative analysis of online forum discussions on cannabis and ADHD. PLoS One.

[53] Mitchell JT, Weisner TS, Jensen PS, Murray DW, Molina BSG, Arnold EL, Hechtman L, Swanson JM, Hinshaw SP, Victor EC, Kollins SH, Wells KC, Belendiuk KA, Blonde A, Nguyen C, Ambriz L, Nguyen JL (2017). How substance users with ADHD perceive the relationship between substance use and emotional functioning. J Atten Disord.

[54] Mayer KH, Chan PA, Flash CA, Krakower DS (2018). Evolving models and ongoing challenges for HIV pre-exposure prophylaxis implementation in the United States. J Acquir Immune Defic Syndr.

[55] Kelley CF, Kahle E, Siegler A, Sanchez T, Del RC, Sullivan PS, Rosenberg ES (2015). Applying a PrEP continuum of care for men who have sex with men in Atlanta, Georgia. Clin Infect Dis.

[56] Abaasa A, Hendrix C, Gandhi M, Anderson P, Kamali A, Kibengo F, Sanders EJ, Mutua G, Bumpus NN, Priddy F, Haberer JE (2018). Utility of different adherence measures for PrEP: patterns and incremental value. AIDS Behav.

[57] Baker Z, Javanbakht M, Mierzwa S, Pavel C, Lally M, Zimet G, Gorbach P (2017). Predictors of over-reporting HIV pre-exposure prophylaxis (PrEP) adherence among young men who have sex with men (YMSM) in self-reported versus biomarker data. AIDS Behav.

[58] Koss CA, Hosek SG, Bacchetti P, Anderson PL, Liu AY, Horng H, Benet LZ, Kuncze K, Louie A, Saberi P, Wilson CM, Gandhi M (2018). Comparison of measures of adherence to human immunodeficiency virus preexposure prophylaxis among adolescent and young men who have sex with men in the United States. Clin Infect Dis.

[59] Albert W, Tullis T (2013). Measuring the user experience: collecting, analyzing, and presenting usability metrics. 2nd ed.

[60] Tullis T, Stetson J A comparison of questionnaires for assessing website usability.

[61] Petry NM, Martin B (2002). Low-cost contingency management for treating cocaine- and opioid-abusing methadone patients. J Consult Clin Psychol.

[62] Petry NM, Martin B, Cooney JL, Kranzler HR (2000). Give them prizes, and they will come: contingency management for treatment of alcohol dependence. J Consult Clin Psychol.

[63] Petry NM, Martin B, Simcic F (2005). Prize reinforcement contingency management for cocaine dependence: integration with group therapy in a methadone clinic. J Consult Clin Psychol.

