# Supplementary material for: Smartphone-Based Contingency Management Intervention to Improve Pre-Exposure Prophylaxis Adherence: Pilot Trial
Source: JMIR Mhealth Uhealth. 2018 Sep 10;6(9):e10456. doi: 10.2196/10456 (PMC6231728; doi:10.2196/10456)
Supplement: Multimedia Appendix 1 [file mhealth_v6i9e10456_app1.pdf]

Multimedia Appendix 1.

Participants were asked by an examiner to log into mSMART on their phone. From the main home screen, they were asked to complete the following tasks. They were asked to return to the home screen after completing each of the six tasks.

1. Show me where you go on the app to take a picture of your Truvada pill.
2. Change the time of your daily medication reminder.
3. Check to see how much money you've earned using mSMART.
4. Check to see if you have any questions on the SMART Desk.
5. Check to see if upset stomach is a side effect of using Truvada
6. Related to the instruction above, what percentage of people who take Truvada experience an upset stomach at some point?

Supplement Table A. Exit interview task performance

Exit Interview Task	# of seconds<br>M (SD)	# of prompts
1	2.93 (.81)	0
2	6.09 (2.69)	0
3	5.77 (5.53)	0
4	3.90 (1.72)	0
5	6.74 (4.33)	0
6	6.88 (2.52)	0

Notes. Overall mean across tasks was 5.39 (32.31/6)
