# Supplementary material for: Smartphone-Based Contingency Management Intervention to Improve Pre-Exposure Prophylaxis Adherence: Pilot Trial
Source: JMIR Mhealth Uhealth. 2018 Sep 10;6(9):e10456. doi: 10.2196/10456 (PMC6231728; doi:10.2196/10456)
Supplement: Multimedia Appendix 2 [file mhealth_v6i9e10456_app2.pdf]

Multimedia Appendix 2

Supplement Table B. Qualitative category endorsement rates

Qualitative Category	Endorsement Rate
1. mSMART features	
Liked	20%
Disliked	0%
Mixed (liked and disliked features)	80%
2. Daily use	
Facilitator	40%
Barrier	0%
Mixed (facilitators and barriers)	60%
3. mSMART aesthetics	
Liked	10%
Disliked	40%
Mixed (liked and disliked aesthetics)	40%
4. Learning how to use mSMART	
Easy	80%
Difficult	0%
Mixed (easy and difficult)	10%
5. Features of mSMART that should be modified	90%
6. Likelihood of using mSMART depends on how soon you start PrEP or if you have adherence problems	90%
Notes. Categories 3, 4, 5, and 6 were not commented on by one participant (therefore the sum endorsement rate is 90% instead of 100%).	
